# Technical-scientific experience in times of Covid-19: the challenges faced by nursing technicians

**DOI:** 10.15649/cuidarte.4337

**Published:** 2025-04-23

**Authors:** Jordana da Silva Souza, Mariana Crissângila Trigueiro da Silva, Wilma Tatiane Freire Vasconcelos, João André Tavares Álvares da Silva, Josilene de Melo Buriti Vasconcelos, Ana Cristina de Oliveira e Silva, Jocelly de Araújo Ferreira

**Affiliations:** 1 University of Pernambuco (UPE), Recife, Brazil. jordanasouza57@gmail.com University of Pernambuco (UPE) University of Pernambuco (UPE) Recife Brazil jordanasouza57@gmail.com; 2 Federal University of Paraíba (UFPB), João Pessoa, Brazil. trigueiromari@gmail.com Federal University of Paraíba (UFPB) Federal University of Paraíba (UFPB) João Pessoa Brazil trigueiromari@gmail.com; 3 Federal University of Paraíba (UFPB), João Pessoa, Brazil. wilmafreire23@gmail.com Federal University of Paraíba (UFPB) Federal University of Paraíba (UFPB) João Pessoa Brazil wilmafreire23@gmail.com; 4 School of Public Health of the State of Minas Gerais (ESP), Belo Horizonte, Brazil. joao.silva@esp.mg.gov.br School of Public Health of the State of Minas Gerais (ESP) School of Public Health of the State of Minas Gerais (ESP) Belo Horizonte Brazil joao.silva@esp.mg.gov.br; 5 Lecturer from the Federal University of Paraíba (UFPB), João Pessoa, Brazil. josilenedemelo@gmail.com Federal University of Paraíba (UFPB) Federal University of Paraíba (UFPB) João Pessoa Brazil josilenedemelo@gmail.com; 6 Lecturer from the Federal University of Paraíba (UFPB), João Pessoa, Brazil. anacris.os@gmail.com Federal University of Paraíba (UFPB) Federal University of Paraíba (UFPB) João Pessoa Brazil anacris.os@gmail.com; 7 Lecturer from the Federal University of Paraíba (UFPB), João Pessoa, Brazil. jocellyaferreira@hotmail.com Federal University of Paraíba (UFPB) Federal University of Paraíba (UFPB) João Pessoa Brazil jocellyaferreira@hotmail.com

**Keywords:** Nursing, Covid-19, Intensive Care Units, Enfermería, Covid-19, Unidades de Cuidados Intensivos, Enfermagem, Covid-19, Unidades de Terapia Intensiva

## Abstract

**Introduction:**

The Covid-19 pandemic has posed a significant challenge to public health worldwide, leading to the collapse of healthcare institutions and professionals, particularly nursing technicians, who provide direct patient care.

**Objective:**

To investigate nursing technicians' perceptions of their technical-scientific experiences while caring for Covid-19 patients.

**Materials and Methods:**

An exploratory and descriptive study with a qualitative approach was conducted in a university hospital. The Iramuteq software was used to analyze the data, applying the Descending Hierarchical Classification Method alongside Bardin's Content Analysis Technique.

**Results:**

It was identified that the participants' technical-scientific skills are associated with previous ICU experience and active participation in training sessions, both facilitating factors for performing the procedures. Additionally, the negative effects of care practices were observed, leading to physical and mental exhaustion.

**Discussion:**

Work overburden, fear of contagion, high mortality rates, and social isolation were identified as causes of stress and mental exhaustion among professionals, making resilience a crucial factor in adapting to these adversities.

**Conclusion:**

Identifying the nursing professionals' prior knowledge is an important tool for assessing the quality of care provided and the professionals' safety in care delivery. Moreover, implementing health policies that offer psychological support for health professionals is essential.

## Resumen

**Introducción:**

La pandemia de Covid-19 se ha convertido en un desafío para la salud pública mundial, provocando el colapso de las instituciones de salud y de los profesionales, especialmente los técnicos de enfermería, que atendían directamente a los pacientes.

**Objetivo:**

Investigar la percepción de los técnicos de enfermería sobre la experiencia técnico-científica durante la atención a pacientes con Covid-19.

**Materiales y métodos:**

Estudio exploratorio y descriptivo, con enfoque cualitativo, realizado en un hospital universitario. Para el análisis de los datos se utilizó el software Iramuteq, a través del Método de Clasificación Jerárquica Descendente en asociación con las Técnicas de Análisis de Contenido, descritas por Bardin.

**Resultados:**

Se identificó que la aptitud técnico-científica de los participantes está asociada a la experiencia previa en UCI y a la participación efectiva en los entrenamientos, constituyendo factores facilitadores para la realización de los procedimientos. Además, se observan impactos negativos derivados de las prácticas de cuidado, que generan desgaste físico y mental.

**Discusión:**

La sobrecarga de trabajo, el miedo al contagio, las altas tasas de mortalidad y el aislamiento social fueron identificados como causas de estrés y agotamiento mental por los profesionales, haciendo de la resiliencia un factor crucial para la adaptación frente a estas adversidades.

**Conclusiones:**

Identificar los conocimientos previos de los profesionales de enfermería es una herramienta importante para evaluar la calidad de la atención prestada, así como la confianza de los profesionales en llevarla a cabo. Además, es fundamental implementar políticas de salud que brinden apoyo psicológico a los profesionales de la salud.

## Resumo

**Introdução:**

A pandemia de Covid-19 se tornou um desafio para a saúde pública mundial, ao levar instituições e profissionais de saúde ao colapso, destacando-se os técnicos de enfermagem, que estiveram nos cuidados diretos aos pacientes.

**Objetivo:**

Investigar a percepção dos técnicos de enfermagem a respeito da vivência técnico-científica durante a assistência ao paciente com Covid-19.

**Materiais e métodos:**

Estudo exploratório e descritivo, com abordagem qualitativa, realizado em um hospital universitário. Para análise dos dados, foi utilizado o software Iramuteq, através do Método da Classificação Hierárquica Descendente, em associação com a Técnica de Análise Conteúdo descrita por Bardin.

**Resultados:**

Identificou-se que a aptidão técnico-científica dos participantes está associada a experiência prévia em UTI e a participação efetiva em treinamentos, constituindo-se em fatores facilitadores para a realização dos procedimentos. Ainda, observam-se os impactos negativos advindos das práticas assistenciais, que geraram desgastes físicos e mentais.

**Discussão:**

A sobrecarga de trabalho, o medo da contaminação, as altas taxas de mortalidade e o isolamento social foram apontados como causadores de estresse e esgotamento mental pelos profissionais, tornando a resiliência um fator crucial para adaptação diante dessas adversidades.

**Conclusões:**

Identificar o conhecimento prévio dos profissionais de enfermagem constitui uma ferramenta importante para avaliar a qualidade da assistência prestada, bem como a segurança dos profissionais em executá-la. Além disso, é fundamental que haja a implementação de políticas de saúde que deem suporte psicológico para os profissionais de saúde.

## Introduction

Although the epidemiological situation of Covid-19 in Brazil is currently stable^1^, it is estimated that by December 2022, it had caused more than 6.6 million deaths worldwide. In Brazil, official records indicate that 690,000 people lost their lives due to complications from this disease, while the number of people infected is 35.4 million^2^. 

Due to its high transmissibility, the rapid advance of the coronavirus in the country led to a significant increase in the demand for healthcare services to treat these patients. This demand was reflected in primary care services and, mainly, in high-complexity services such as intensive care units for severe cases^3^. 

Brazil has the Unified Health System (SUS, for its acronym in Portuguese), established by the Federal Constitution of 1988 in response to popular demands, offering free and universal healthcare^4^. Nevertheless, the effective implementation of the SUS continues to face financial and administrative challenges, which have been exacerbated by neoliberal underfunding and privatization policies that have prioritized limiting the state's role since the 1990s^5^. The Brazilian fiscal crisis was further exposed by the 2016 spending cap, and the Covid-19 pandemic revealed the fragility of the Brazilian healthcare system^6^. 

However, even before the pandemic, the insufficiency of Intensive Care Unit (ICU) services in the SUS was already evident, with the number of available beds falling significantly below recommended standards. In addition, inequalities in bed distribution across regions were evident, such as the North and Northeast, which had the lowest bed-to-population ratio^7^. 

During the pandemic, in order to meet urgent healthcare needs, Brazil expanded the number of ICU beds available through the SUS, which previously had 35,682 beds, by adding 17,240 new beds exclusively for Covid-19^8^. However, this expansion was insufficient to meet the demand, leading to ICU overcrowding, supply shortages, and a lack of specialized equipment, infrastructure, and beds^9^. 

The unequal distribution of ICU beds led to the collapse in the states of Amazonas, Pernambuco, and Ceará, where ICU bed occupancy for Covid-19 reached 100%. In Amazonas, the situation was even more critical, with a completely overwhelmed health system, resulting in a high number of deaths. The lack of space to store the bodies necessitated the use of refrigerated containers to accommodate the victims, as well as the opening of mass graves for burials^4^. 

It is evident that establishing and maintaining ICU beds require substantial investments in infrastructure and the recruitment of a skilled workforce^7^. Among the professional groups working in these units, nursing stands out. 

The work of nursing professionals during the pandemic heightened recognition of the importance of nursing care, which supports the patient throughout all stages of the health-disease process, from admission to discharge or death^10^. Within this team, nursing technicians constitute the largest segment of the workforce and spend most of their time with patients, providing direct care. 

Nevertheless, the pandemic created an urgent need for nursing to update knowledge in intensive care, as protocols for managing critically ill patients in ICUs change constantly with new discoveries^11^. These professionals had to deal with changes in care routines, including compliance with infection prevention measures, precautionary measures, protective clothing, reduced bed bathing time, patient repositioning, and other essential care activities^12^.

Nonetheless, frontline nursing professionals were also exposed to several stressors, including long working hours, physical and emotional strain, as well as negative emotions related to feelings of danger, uncertainty, or anger. Consequently, caring for Covid-19 patients was associated with insomnia, fatigue, and various psychological disorders, such as anxiety, depression, and burnout^13^,^14^,^15^. Studies indicate that all these factors not only interfere with work performance but also comprise patient safety and the quality of care^14^,^16^. 

Therefore, it is essential that the care of critically ill patients is provided by a team trained to manage this condition^17^. Although several studies have addressed the experiences of nursing professionals during the Covid-19 pandemic, scientific production on their technical and scientific experience remains scarce. 

Accordingly, it is important to explore whether nursing is prepared to perform procedures on critically ill Covid-19 patients, particularly through mid-level professionals responsible for direct care. Therefore, the objective of this study is to investigate the nursing technicians' experiences regarding their technical and scientific skills in the care of Covid-19 patients. 

## Materials and Methods

This is a field study with a qualitative approach, employing an exploratory and descriptive design as part of a broader research project. The research was conducted using convenience sampling between August and September 2022 at a university hospital in João Pessoa —PB. 

The study population included nursing technicians who provided intensive care to Covid-19 patients. These professionals were part of the institution's active and permanent staff. They were selected based on the ICU work schedules in effect during the period of care for Covid-19 patients. 

It is noteworthy that this study was not confined to a single hospital service, as some professionals were reassigned from or originally belonged to other services but were involved in providing intensive care to Covid-19 patients. 

The inclusion criteria comprised nursing technicians who were part of the hospital’s permanent staff, worked in the adult Covid-19 ICU, and had directly cared for patients with Covid-19. The following criteria were considered to exclude the participants: nursing technicians with a physical or mental condition that made it difficult to understand the interview. 

To initiate data collection, a search was conducted using the nursing schedules of the Covid-19 ICU, which were available on the institution’s website and covered its period of operation between 2020 and 2021. Based on these schedules, the listed professionals were identified, and in collaboration with the service coordinator, we selected the technicians who met the established inclusion criteria. Subsequently, we contacted the selected professionals to inform them about the objectives of this research and the procedures for conducting the interviews and to extend an invitation for voluntary participation, ensuring anonymity and requiring the signing of the consent form. In total, 12 nursing technicians agreed to participate, thus forming the study sample. 

Empirical data were collected through an open interview guided by a script and audio recorded using a cell phone, followed by transcription. For professionals whose work schedules made face-to-face meetings unfeasible, remote interviews were conducted via internet streaming, a method used by two participants. 

The participants answered the following questions: “Can you describe the difficulties and facilitating factors in performing nursing care?” and “Based on what you have already told me, did you feel or do you feel technically and scientifically prepared to provide this care?” The interview location was chosen by the participants, so that they felt more comfortable and safer, and they varied between designated rest areas, hospital corridors, or their respective services. 

To analyze the results, Bardin’s Content Analysis Technique^18^ was applied in conjunction with the Iramuteq software. This process was carried out in five stages. 

The first stage consisted of processing the material, during which the interview content was transcribed and formatted for compatibility with the software before being imported. 

In the second stage, the interview transcripts were imported into Iramuteq (version 0.7 alpha 2) for analysis. The Descending Hierarchical Classification (DHC) method was applied, which analyzes the text segments and classifies them based on their vocabulary. These texts were divided into groups and classified according to the frequency of lemmatized forms using chi-square tests to check the association between words and segments^19^. 

In the third stage, an analysis was conducted following Bardin’s technique^18^, aiming to identify relevant categories and indicators based on processes described by the author: pre-analysis, exploration of the material, treatment of results, inference, and interpretation. At this stage, the transcribed texts were skimmed to become familiar with the data and define the preliminary categories. 

In the fourth stage, the material was systematically examined, considering the data generated by Iramuteq, which was presented as a dendrogram. The units were grouped into categories and subcategories. In the final stage, the data were processed and qualitatively interpreted, identifying thematic patterns and relationships between the categories. 

 The DHC analysis guided the identification of the main thematic categories. This quantitative approach to data analysis served as a basis for organizing and qualitatively interpreting the findings, enabling data triangulation. It is noteworthy that no validation process was conducted with the study participants. The dataset was stored in Mendeley Data for free access and consultation^20^.

Regarding ethical considerations, this study was conducted in accordance with the recommendations of Resolution nº 466/12 of the Brazilian National Health Council (CNS, for its acronym in Portuguese) and received approval under opinion number 5.482.113. 

## Results

The sample consisted of 12 nursing technicians. Based on the socio-professional profile of the participants, the majority were females (58.33%), aged 30 to 40 years (83.33%), and married (58.30%). As for occupational characteristics, the most common length of education was 16 to 19 years (50.00%), and less than 5 years of experience in ICUs (50.00%). Additionally, 50.00% of the professionals held a higher education degree in nursing. 

Regarding the experiences reported by the nursing technicians, the Reinert Method for Descending Hierarchical Classification (DHC) was applied to analyze the statements. The corpus of this study comprised 12 texts containing 304 analyzed text segments, with 80.26% of the corpus used. The analysis identified 10,590-word occurrences across 1,604 distinct forms. 

**Figure 1:**
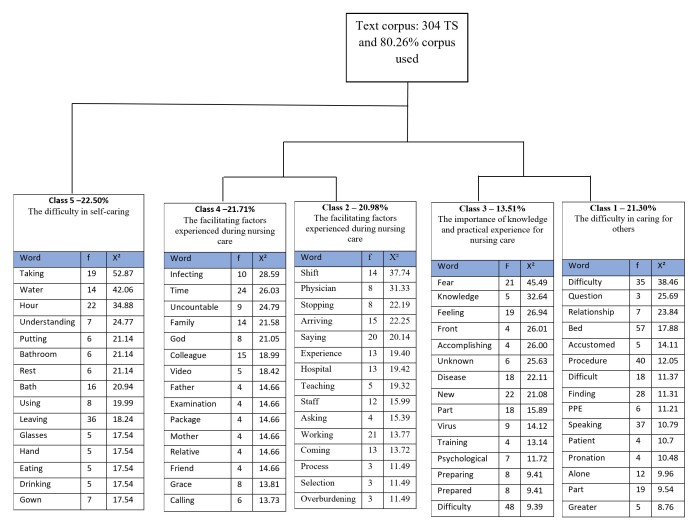
Descending Hierarchical Classification (DHC) – Dendrogram*

*Source: Adapted by the authors based on DHC analysis using Iramuteq (2022). All word occurrences with p <0.05 were included. *

From the corpus analysis, five classes emerged, which are presented in the dendrogram generated by Iramuteq (Figures 1). In this dendrogram, the classes are divided into two main branches: Branch 1 leads to Class 5, while Branch 2 gives rise to the remaining classes (1, 2, 3, and 4). 

Based on the classes derived through DHC and analyzed using Bardin’s Content Analysis Technique^18^, three thematic categories were identified, namely: Difficulties encountered in performing nursing procedures, which is subdivided into two subcategories (difficulties in self-caring [Class 5] and difficulty in caring for others [Class 1]); facilitating factors experienced in nursing care (Classes 2 and 4), and the importance of knowledge and practical experience in nursing care (Class 3). All classes consisted of words with a χ2 test coefficient above 3.84, corresponding to p < 0.05, representing the significance threshold in Iramuteq^19^. 

**Category 1 –Difficulties encountered in performing nursing procedures**

This category refers to nursing technicians' accounts of the difficulties they experienced while performing procedures in the ICU to meet the health needs of critically ill Covid-19 patients. Given the scope of this theme, two subcategories emerged, as described below. 

** Subcategory 1 –Difficulties in caring for others**

This subcategory corresponds to Class 1, which accounts for 21.31% (f=52 TS) of the total analyzed corpus. It includes words and roots within a χ2 coefficient range of 8.76 (highest) to 38.46 (difficulty). This subcategory comprises words such as “Difficulty” (χ2>38.46), “Bed” (χ2>17.88), “Procedure” (χ2>12.05), “Difficult” (χ2>11.37), “PPE” (χ2>11.21), “Patient” (χ2>10.7), “Pronation” (χ2>10.48) and “Accustomed” (χ2>14.11). 

When analyzing data, it was found that the reports showing technical skills for performing intensive nursing procedures on Covid-19 patients were grouped based on their similarity to routine procedures already established in a general ICU. However, despite being common procedures, the severity of these patients' clinical conditions made their execution more difficult for professionals.

“[...] Although they were critically ill patients that we were already accustomed to caring for, intensive care patients, these patients were far more severely ill than what we were accustomed to”. (E12)

“[...] Bed bathing was itself another difficulty due to the severity of pronated patients; so, it was a great difficulty”. (E10)

Although these procedures were similar, professionals also had to adapt to the prone positioning maneuver, a therapy used to treat Covid-19 patients that was rarely implemented in ICU routines before the pandemic.

“[...] Pronation itself was another difficulty, as it was not a routine procedure and required extra caution from professionals during the patient mobilization”. (E10)

In their statements, the nursing technicians described the exhausting work routine they faced during the pandemic, characterized by an increased demand for care due to the severity of patients' clinical conditions, which required more nursing hours. It is noteworthy that, driven by a reduction in the number of professionals, many of them were sidelined due to Covid-19-related illness or pre-existing comorbidities.

“[...] So, like this, the work was greater. The time, the workload was the same, but the work itself was different, it was very different”. (E1)

“[...] We had set entry times, but sometimes no clear exit times. We had to dress, undress, shower, have lunch, and then go right back in—there were no breaks, no rest”. (E11)

**Subcategory 2 – Difficulties in self-caring**

This subcategory corresponds to Class 5, accounting for 22.54% (f=55 TS) of the total analyzed corpus. It includes words and roots within a χ2 coefficient range of 52.87 (Taking) to 17.54 (Gown, Putting, Bathroom, Rest, Glasses, Hand, Eating, Drinking). It consists of words such as “Water” (χ2> 42.06), “Bathroom” (χ2> 21.14), “Glasses” (χ2> 17.54), “Eating” (χ2>17.54), “Gown” (χ2>17.54), and “Leaving” (18.24). 

These words were found to be associated with the pandemic scenario caused by Covid-19, which introduced a new reality to ICU procedures, requiring attention to self-care due to the disease's transmissibility. In their reports, nursing technicians mentioned the use of Personal Protective Equipment (PPE) as a challenge they faced, given the prolonged hours they had to wear them continuously, which caused harm to these professionals’ health.

“[...] In the beginning, the biggest difficulty was the protective clothing. We had to wear those gowns and couldn’t go out to urinate or do anything else. We would stay inside for about six hours before being relieved. We only went to the bathroom after those six hours”. (E5)

“[...] countless times we went without being able to pee, many of my female colleagues experienced recurrent urinary tract infections”. (E2)

Given the transmissible nature of the disease, which marked the work experience of healthcare professionals due to the constant risk of Covid-19 contagion, nursing technicians’ reports highlight an intense fear of falling ill—both for themselves and, more importantly, for their family members. The possibility of being in the same critical condition as the patients they were caring for created a distressing scenario, which, in turn, was exacerbated by the lack of knowledge about the disease, as the evolving and often imprecise information was still being studied by researchers worldwide.

“[...] since it was an unknown virus, we had no way to know whether we could be in the ICU one day and contract it the next—or what might or not happen to us”. (E2)

As for the risk of infecting their family members, some professionals mentioned the need for physical distancing as a protective measure from the virus.

“[...] Many colleagues at some point could not bear to go home and possibly infect their parents. Some even had to distance themselves from their relatives”. (E2)

“[...] I didn’t see my father for about three or four months because I was afraid of passing it to him”. (E4)

The reports evidenced a process of psychological distress resulting from the experiences professionals faced in the ICU. This suffering was mainly attributed to the high number of deaths these professionals had to witness and manage.

“[...] As much as we try to prepare ourselves for the moment of death, no one could have been prepared for what we saw in there. Losing many patients in a single shift was deeply unsettling for many people. Personally, I can say that there were psychological sequels after the pandemic.” (E10)

“[...] We didn’t know what was happening outside, all we saw were patients coming and going, many deaths, and we didn’t have this psychological part”. (E7)

“[...] But I can’t see myself in the next pandemic, it’s so much that I told myself that once this pandemic was over, I would study to leave this field, because I didn’t want to see people dying anymore.” (E11)

Regarding the psychological distress experienced, one participant highlighted the lack of institutional support, noting that while the focus was on patients' needs, there was little sensitivity toward the professionals providing care. Thus, difficulties related to administrative management are evidenced.

“[...] I think they were all focused on providing the best care for patients, but they left healthcare professionals aside. Professionals also need care but this often goes unnoticed by those in charge”. (E10)

**Category 2 –Facilitating factors experienced during nursing care**

This subcategory corresponds to Classes 2 and 4, accounting for 20.9% (f=51 TS) and 21.72% (f=53 TS) of the analyzed corpus, respectively. In Class 2, words fell within a χ2 coefficient range of 11.49 (Selection process, Overburden) to 37.74 (Physician). Similarly, in Class 4, words were within a χ2 range of 13.73 (Calling) to 28.59 (Infecting). The keywords in this subcategory include “Infecting” (χ2> 28.59), “Selection process” (χ2>11.49), “Teaching” (χ2>19.32), “Experience” (χ2>19.4), “Shift” (χ2> 37.74), “Working” (χ2> 13.77), “Family” (χ2> 21.58), “God” (χ2>21.05) and “Package” (χ2 >14.66). 

The analysis of the reports evidenced the presence of facilities for nursing care provided by nursing technicians. The professionals' accounts are centered on two interpretations. The first pertains to participants for whom negative experiences outweighed positive ones and, consequently, they were unable to recall aspects that had positively influenced patient care, thereby reinforcing the difficulties already mentioned. For these participants, the following excerpts are particularly noteworthy:

“[...] There were no facilitating factors, there were zero. For me, it was the worst experience of my life. It was very difficult; it was truly difficult. You arrived at a shift where no one knew what to do, the physicians didn’t know how to treat the patients, they were afraid, the fear on their faces was visible”. (E6)

The second interpretation includes participants who, despite all the adversities, were able to recall, albeit in isolation, positive aspects that they felt facilitated patient care. Among these aspects, the previous experience in intensive care, team cohesion, and the material support provided by the hospital stand out.

“[...] I would say that facilitating factors came from experience in treating ICU patients. Since I already had experience, not just from here, but in other ICU hospitals; for me, that was the greatest facility”. (E10)

“[...] I think the facilitating factors we had here were access to medication and PPE. From the beginning we were provided with gowns and masks, so in that regard, we were always well-equipped, you know? I think the facilitating factors were those”. (E4)

“[...] The positive thing is that the team became very close, we were truly united. The hours we spent outside, like lunchtime, we fraternized and stayed together. Moreover, whenever one of us felt like giving up, someone else was there to held their hand”. (E1)

Nonetheless, although these participants unanimously pointed out the facilitating factors, they also recalled the difficulties they experienced, reaffirming their negative impact. Among these difficulties was the hospital's selection process opened for hiring new professionals to work in the Covid-19 ICU. This selection process, which should ease the professionals' workload, was perceived by the participants as a complicating factor due to the inexperience and lack of knowledge among newly hired staff.

“[...] we thought that they would conduct the selection process and choose people with ICU experience. However, there were people who came here, who had only worked in the PSF (Family Health Program in Brazil) and were assigned to the ICU”. (E3)

“[...] there were people who came here, who had only worked in the PSF (Family Health Program in Brazil) and were assigned to the ICU. Besides worrying about our own health and avoiding contagion, we had to redouble cautions with critically ill patients because we couldn’t fully trust these newly hired people. They lacked experience in caring for critical patients and preparing medications”. (E2)

The nursing technicians also reported that, despite the need, their work routine did not allow them to train the newly hired professionals. As alternative, these professionals were initially assigned to the general ICU for a period, allowing them to adapt to routine procedures in the general ICU before being reassigned to the Covid-19 ICU.

“[...] we asked them to leave, go to the general ICU to train and then return there. This is because you couldn’t do two things like that. You either worked or taught”. (E3)

**Category 3 – The importance of knowledge and practical experience for nursing care**

 This category corresponds to Class 3, which accounts for 13.52% (f=33 TS) of the analyzed corpus, and the words fall within the χ2 coefficient range from 9.39 (Difficulty) to 45.49 (Fear). In this category, the most significant words include “Knowledge” (χ2>32.64), “Training” (χ2>13.14), “Accomplish” (χ2>26.0), “Unknown” (v>25.63), “Prepared” (χ2>9.41), “New” (χ2>21.08), and “Disease” (χ2>22.11).

Based on this analysis, it was possible to identify reports in which the participants were asked whether they felt technically and scientifically prepared to work in the Covid-19 ICU, and they precisely responded that they were able to provide such care. The experience and training before the pandemic were decisive in fostering this sense of preparedness.

“[...] It was a new disease, but we were prepared. We knew that we were facing something new, yet all the procedures would, in some way, be similar to what we had already been through.” (E2)

“[...] I felt prepared because I had been working in the ICU for a long time. Plus, we had a sort of training session before, but the training was only about the protective clothing.” (E5)

“[...] we were truly (prepared) because, before working in the Covid-19 ICU, we received training, specific training for Covid-19.” (E9)

Conversely, two of the participants reported that they did not feel prepared, as they perceived not having enough training to meet the demands of patients in a Covid-19 ICU.

“[...] We had very little, little training. Therefore, I don’t think anyone was prepared [...] for the pandemic itself, how to respond, prevent infections, or take all the precautions. We came to learn this routine during the course of the pandemic”. (E3)

“[...] Was I prepared? No, I wasn’t. I just followed the required protocol, things, routines. I wasn’t in the frame of mind to study, precisely because of this issue, knowing that people weren’t making it out”. (E11).

## Discussion

 The analysis of the results provided insight into the technical-scientific experiences of nursing technicians in response to the new demand for intensive procedures for Covid-19 patients. It was also possible to identify the difficulties arising from performing these procedures. These findings corroborate studies that identified service overload, exhausting work routines, prolonged use of PPE, and mental distress^21^,^22^ as challenges faced by nursing professionals working in ICUs and providing direct care to Covid-19 patients.

 The interaction with an unknown and highly transmissible disease, combined with exposure to death due to its high lethality rate —comparable to that of a war scenario—transformed these professionals’ work routines into a daunting environment demanding intense physical and mental effort^23^.

 Although indispensable, using PPE was not enough to foster a sense of safety and protection among workers. It is worth mentioning studies that describe the physical discomfort caused by prolonged use of this equipment, leading to skin lesions, physical fatigue, and a significant decline in work performance. Additionally, PPE use restricted professionals from attending to their physiological needs^23^.

 A study conducted in Spain^24^ exploring the experiences of intensive care nurses caring for Covid-19 patients identified fear as the most prevalent emotion. According to this study, the fear expressed by the participants influenced their experience during the pandemic, leading to changes in their daily work routines, reduced sleep quality, and a negative impact on patient care.

 In addition, the fear of transmitting the virus to their family made some professionals distance themselves physically. This phenomenon is also reported in other studies, which mentioned the efforts of nurses to protect their family members by staying in temporary accommodations, sometimes provided by the hospital. Nevertheless, adopting these measures was often accompanied by feelings of loneliness and isolation^22^.

 According to a systematic review^25^, nurses are the health professionals who are most vulnerable to mental health disorders and register the highest prevalence of Burnout Syndrome. This condition can be associated with low wages, long working hours, and excessive workloads, factors further intensified by the pandemic and exacerbated by traumatic events resulting from this scenario.

 A study conducted in Canada points out that within the nursing team, professionals serving as team nurses are more vulnerable to developing post-traumatic stress symptoms^26^. When analyzed in light of the Brazilian reality, nursing technicians are most at risk for developing these symptoms due to the technical division of labor, which requires them to maintain direct and prolonged contact with patients.

 In this scenario of intense stress and mental exhaustion, a study indicates that healthcare professionals resorted to self-management strategies to mitigate the emotional impacts of the pandemic. Among the employed strategies were increased focus on work, reducing exposure to Covid-19 news, engaging in leisure activities such as watching movies, reading, and taking baths, as well as maintaining good nutrition and rest^27^.

 Participants refer to similarities between high-complexity procedures for Covid-19 and non-Covid-19 patients. Nevertheless, the severity of the clinical condition in Covid-19 patients was the main difference between care procedures, corroborating the findings of another study^28^. Despite procedural similarities, Covid-19 patients often experience sudden and unpredictable changes in their breathing patterns, leading to a rapid decline in health and requiring increased attention and nursing time^29^. As a consequence, caring for Covid-19 patients increases the nursing workload.

 As the pandemic progressed, the rising number of critically ill patients requiring intensive care led to a shortage of skilled workforce, a crisis further exacerbated by the pandemic. Thus, the growing demand for professionals was met by nurses without proper ICU training^30^. The participants in this study identified the recruitment of inexperienced ICU professionals as a challenge, given the constant need to train these professionals, who lacked the necessary skills for the role.

 One study^21^ highlights that nursing professionals with ICU competencies had to take on leadership roles and support less experienced professionals, increasing their workload. Working alongside professionals without the necessary skills to deliver intensive care became a source of moral distress for experienced nurses^31^.

 The results of this study evidenced that effective participation in hospital-provided training and courses was decisive for nursing technicians to feel competent in providing the necessary care to Covid-19 patients. Nevertheless, the pandemic exacerbated stress, time constraints, and physical and mental exhaustion, which posed barriers to participation in educational activities^32^.

 Regarding the ability of professionals to identify positive aspects of the intensive care provided to Covid-19 patients, only half of the participants reported positive aspects. It is noted that workplace resilience provides workers with ways to generate more positive responses to adverse situations. A study indicates that resilience, which often emerges in adverse or disaster scenarios, is inversely proportional to mental disorders such as anxiety and depression^33^.

 Research indicates that frontline professionals responding to Covid-19 exhibit the lowest resilience scores, given the challenging conditions they face^33^. Among healthcare workers, nursing professionals report the lowest resilience scores, and within the nursing team, nursing technicians have the lowest resilience rates^34^.

 Among the limitations of this study, it is important to note that it was conducted in a single setting, with participants who have specific social and economic characteristics and whose experiences may not represent the reality of the category in the country, thus limiting generalizability. Another limitation of the study was the time frame of data collection –months after the nursing technicians had ceased providing care to Covid-19 patients. This timing may have made the participants more susceptible to individual changes related to memory and stress experienced under those circumstances.

## Conclusions

The findings of this study evidenced the technical skills of nursing technicians at this hospital institution in response to the new demands of intensive care procedures for Covid-19 patients. However, while it represents an important finding, it cannot be generalized, as the data were collected from a group of professionals within a specific healthcare institution. 

It was found that participation in institutional training was essential for nursing technicians to feel competent in performing their care activities. Conversely, interaction with professionals lacking technical skills in intensive care posed a challenge that led to an increased workload on more experienced professionals. 

From the participants’ accounts, it was possible to identify the negative impacts of their work activities, which led to physical and psychological exhaustion. Fear stood out as a theme in the professionals’ reports, as they expressed concern for their own health and that of their families. 

These findings highlight the need for psychosocial support strategies for nursing professionals who serve on the front lines, providing intensive care to critically ill coronavirus-infected patients, as well as the importance of further research on the topic. 

## References

[1] 1. Santos PPGV dos, Oliveira RAD de, Albuquerque MV de. Desigualdades da oferta hospitalar no contexto da pandemia da Covid-19 no Brasil: uma revisão integrativa. *Saúde em Debate.* 2022;46(1):322-37. 10.1590/0103-11042022E122

[2] 2. World Health Organization. WHO Coronavirus (Covid-19) Dashboard [Internet] 2022 [cited 2022 dec 08]. Available from: https://covid19.who.int/region/amro/country/br

[3] 3. Avelar FG de, Emmerick ICM, Muzy J, Campos MR. Complications of Covid-19: developments for the Unified Health System. *Physis: Revista de Saúde Coletiva.* 2021;31(1):e310133. 10.1590/S0103-73312021310133

[4] 4. Palamim, CVC; Marson, FAL. Covid-19–The availability of icu beds in Brazil during the onset of pandemic. *Annals of global health. *2020;86(1). 10.5334/aogh.3025 PMC7427679 32864352

[5] 5. Carnut, L; Ferraz, CB. Necessidades em (de) saúde: conceitos, implicações e desafios para o Sistema Único de Saúde. *Saúde em debate.* 2021;45(129):451-466. 10.1590/0103-1104202112916

[6] 6. Rossi, P; Dweck, E. Impactos do novo regime fiscal na saúde e educação. *Cadernos de Saúde Pública.* 2016;32(12):e00194316. 10.1590/0102-311X00194316 27992044

[7] 7. Medeiros RS. Insuficiência de leitos de UTI: crise do capital e mercantilização da saúde. *Argumentum*. 2018;10(1):229-40. 10.18315/argumentum.v10i1.18647

[8] 8. Silva, LL; Dutra, AC; Andrade, L; Iora, PH; Ramajo, GLR; Gualda IAP et al. Emergency care gap in Brazil: geographical accessibility as a proxy of response capacity to tackle Covid-19. *Frontiers in Public Health.* 2021;9:740284. 10.3389/fpubh.2021.740284 PMC8634954 34869155

[9] 9. Zin CFF, Miolo DP, Conci L, Buss E, Prestes NO, Ferrão L, et al. Atuação de acadêmicos de enfermagem em uma unidade de terapia intensiva Covid-19: um relato de experiência. *Brazilian Journal of Health Review.* 2021;4(6):25770-84. 10.34119/bjhrv4n6-175

[10] 10. Paixão GLS, Freitas MI, Cardoso LCC, Carvalho AR, Fonseca GG, Andrade AFSM, et al. Estratégias e desafios do cuidado de enfermagem diante da pandemia da Covid-19. *Brazilian Journal of Development.* 2021;7(2):19125-39. 10.34117/bjdv7n2-521

[11] 11. Souza TM, Lopes GS. Assistência de enfermagem em terapia intensiva ao paciente com Covid 19: um relato de experiência. *Revista Eletrônica Acervo Enfermagem.* 2021; 9: 6118. 10.25248/reaenf.e6118.2021

[12] 12. Gerolin FSF, Pires AM, Nascimento C, Schimitt C, Bucione FTS, Rocha JSA, et al. Ações de lideranças da Enfermagem na organização do atendimento hospitalar a pacientes com Covid-19. *Enfermagem em Foco*. 2020;11(2):207-211. http://revista.cofen.gov.br/index.php/enfermagem/article/view/3665/1007

[13] 13. Zhang Y; Wang C; Pan W; Zheng J; Gao J; Huang X, et al. Stress, burnout, and coping strategies of frontline nurses during the Covid-19 epidemic in Wuhan and Shanghai, China. *Frontiers in psychiatry*. 2020;11:565520. 10.3389/fpsyt.2020.565520 PMC7649755 33192686

[14] 14. Tomaszewska, K; Majchrowicz, B; Snarska, K; Telega, D. Stress and occupational burnout of nurses working with Covid-19 patients. *International journal of environmental research and public health*. 2022;19(19):12688. 10.3390/ijerph191912688 PMC9566059 36231988

[B15] 15. Sagherian, K; Steege, L; Cobb, S.J; Cho, H. Insomnia, fatigue and psychosocial well‐being during Covid‐19 pandemic: A cross‐sectional survey of hospital nursing staff in the United States. *Journal of clinical nursing.* 2023;32(15-16):5382-5395. 10.1111/jocn.15566 PMC7753687 33219569

[B16] 16. Kerlin MP; Mcpeake J; Mikkelsen ME. Burnout and joy in the profession of critical care medicine.* Crit Care.* 2020;24:633-642. 10.1186/s13054-020-2784-z PMC7092567 32204724

[B17] 17. Nunes MR. A atuação do enfermeiro em unidade de terapia intensiva na pandemia de Covid-19: relato de experiência. *Revista Eletrônica Acervo Saúde.* 2020;12(11):e4935. 10.25248/reas.e4935.2020

[18] 18. Bardin L. L’analyse de contenu. Paris. Presses Universitaires de France ; 2013.

[19] 19. Camargo BV, Justo AM. Tutorial para o uso do software de análise textual IRAMUTEQ [Internet]. UFSC; 2013. [citado em 16 de jul 2024]. Disponível em: http://www.iramuteq.org/documentation/fichiers/Tutorial%20IRaMuTeQ%20em%20portugues_17.03.2016.pdf

[20] 20. Souza SS, Silva MTC, Vasconcelos WTF, Silva JATA, Vasconcelos JMB, Silva ACO, et al. A vivência técnico-científica em tempos de Covid-19: os desafios dos técnicos de enfermagem.* Mendeley Data V1*. 2024 10.17632/r8967hhwxj.1

[21] 21. Bergman L, Falk A, Wolf A, Larsson IM. Registered nurses' experiences of working in the intensive care unit during the Covid ‐19 pandemic. *Nursing in Critical Care.Nurs Critic Care.* 2021;26;467-475. 10.1111/nicc.12649 PMC8242789 33973304

[22] 22. Ribeiro JF, Andrade JMF, Melo KAS, Bandeira FLF, Silva PS, Pinho MAB. Profissionais de Enfermagem na UTI e seu protagonismo na pandemia: Legados da Covid-19. *Rev Enferm Contemp*. 2021;10(2);347-365. 10.17267/2317-3378rec.v10i2.3423

[23] 23. Lee SY, Chiang KJ, Tsai KJ, Lin CK, Wang YJ, Chiou CP, et al. Perceived Stress and Coping Behavior of Nurses Caring for Critical Patients with Covid-19 Outbreak in Taiwan: A Mixed-Methods Study. *International Journal of Environmental Research and Public Health*. 2022;19(7):4258. 10.3390/ijerph19074258 PMC8998865 35409938

[24] 24. Moradi Y, Baghaei R, Hosseingholipour K, Mollazadeh F. Challenges experienced by ICU nurses throughout the provision of care for Covid‐19 patients: A qualitative study. *Journal of Nursing Management.* 2021;29(5):1159-68. 10.1111/jonm.13254 PMC8014737 33480145

[25] 25. Fernández‐Castillo RJ, González-Caro M, Fernández-García E, Porcel-Gálvez, AM, Garnacho-Monteiro J. Intensive care nurses' experiences during the Covid ‐19 pandemic: A qualitative study. *Nursing in Critical Care*. 2021;26(5):397-406. 10.1111/nicc.12589 33401340

[26] 26. Gualano MR, Sinigaglia T, Lo Moro G, Rousset S, Cremona A, Bert F, et al. The Burden of Burnout among Healthcare Professionals of Intensive Care Units and Emergency Departments during the Covid-19 Pandemic: A Systematic Review. *International Journal of Environmental Research and Public Health*. 2021;18(15):8172. 10.3390/ijerph18158172 PMC8346023 34360465

[27] 27. Liu Q, Luo D, Haase JE, Guo Q, Wang XQ, Liu S, et al. The experiences of health-care providers during the Covid-19 crisis in China: a qualitative study. *The Lancet Global Health.* 2020;8(6):790-798. 10.1016/S2214-109X(20)30204-7 PMC7190296 32573443

[28] 28. Karanikola M, Mpouzika M, Papathanassoglou E, Kaikoushi K, Hatzioannou A, Leontiou I, et al. Work-Related Traumatic Stress Response in Nurses Employed in Covid-19 Settings. *International Journal of Environmental Research and Public Health.* 2022;19(17):11049. 10.3390/ijerph191711049 PMC9518142 36078761

[29] 29. Sezgin D, Dost A, Esin MN. Experiences and perceptions of Turkish intensive care nurses providing care to Covid‐19 patients: A qualitative study. *International Nursing Review.* 2021;69(3):305-317. 10.1111/inr.12740 34962292

[30] 30. Bambi S, Iozzo P, Lucchini A. New Issues in Nursing Management During the Covid-19 Pandemic in Italy. *American Journal of Critical Care*. 2020;29(4):92-93. 10.4037/ajcc2020937 32467964

[31] 31. Silverman HJ, Kheirbek RE, Moscou-Jackson G, Day J. Moral distress in nurses caring for patients with Covid-19. *Nursing Ethics.* 2021; 28(7-8):1137-1164. 10.1177/09697330211003217 33910406

[32] 32. Nunes MR. A atuação do enfermeiro em unidade de terapia intensiva na pandemia de Covid-19: relato de experiência. *Revista Eletrônica Acervo Saúde.* 2020;12(11):4935-4935. 10.25248/reas.e4935.2020

[33] 33. Vieira LS, Machado WDL, Dal Pai D, Magnago TSB, Azzolin KDO, Tavares JP. Burnout e resiliência em profissionais de enfermagem de terapia intensiva frente à Covid-19: estudo multicêntrico. *Revista Latino-Americana de Enfermagem.* 2022;30:3589. 10.1590/1518-8345.5778.3589 PMC9150431 35649092

[34] 34. Paiva SMA, Silva JCMC, Oliveira MAF, Cardoso MMA. Atuação dos enfermeiros no cuidado de pessoas com transtornos mentais na Estratégia de Saúde da Família. *Revista Eletrônica Acervo Enfermagem. *2021;14:8885. 10.25248/reaenf.e8885.2021

